# A Life Course Perspective on the Sexual Development of Young Intersex People

**DOI:** 10.3390/healthcare12020239

**Published:** 2024-01-18

**Authors:** Mir Abe Marinus, Marianne Cense

**Affiliations:** 1 NNID Netherlands Organisation for Sex Diversity, Staddijk 91, 6537 TW Nijmegen, The Netherlands; 2 Rutgers Sexual and Reproductive Health and Rights, Arthur van Schendelstraat 696, 3511 MJ Utrecht, The Netherlands

**Keywords:** intersex, sexual development, sexual agency, medical treatments, stigma, heteronormativity

## Abstract

Previous research has indicated that intersex people face specific challenges in their sexual development, including uncertainties or confusion about their gender, a negative genital self-image, and hesitance to engage in romantic and sexual relationships. However, in-depth knowledge regarding a central period in this development, adolescence, is missing. In our qualitative study, we explore which factors influence the relational and sexual development of intersex youth and what elements contribute to positive development. We interviewed eighteen intersex persons aged 18–38. We identified three main themes: (1) intersex experiences, (2) the described sexual and relational life course, and (3) factors influencing a positive development. Our findings show that intersex youth face many obstacles in their relational and sexual development, many of which are related to healthcare. However, their life stories also illuminate how healthcare professionals, as well as parents, friends, partners, teachers, and others, can make a substantial difference in intersex lives by breaking normative, binary thinking on sex and gender.

## 1. Introduction

Between 0.05% and 1.7% of humankind is intersex, depending on the definition used (e.g., including only people with either ambiguous genitalia, a genetically confirmed diagnoses, any related diagnosis, or taking a broader approach) and the type of study conducted (medical or a general population study) [1,2,3,4,5,6]. In this article, we will use the term intersex rather than medical notions such as Differences/Disorders in Sex Development (DSD), as we focus on the lived experience of the socio-cultural consequences of being born with a body that does not fit the normative social construct of male and female [7]. Although different forms of sex diversity (including but not limited to Klinefelter syndrome, Turner syndrome, (Complete) Androgen Insensitivity Syndrome, Mayer–Rokitansky–Küster–Hauser syndrome, and Congenital Adrenal Hyperplasia) have various impacts on people’s health and physical well-being, this research focuses on common threads that run through these experiences from a psychosocial perspective. These common threads relate to the social awkwardness, shame, stigma, and secrecy that can accompany any bodily difference coded as “sexual” [8]. Even though challenges for intersex youth are identified in existing research, more specific knowledge about factors that influence their sexual and relational development is minimal. A brief review of literature discussing general life challenges for intersex youth and the sexual and relational development of intersex people is provided below, followed by an introduction to the current study.

Young intersex individuals may face several challenges throughout their youth and adolescence, including insecurities or confusion about their gender, a negative genital self-image, and reluctance to engage in romantic and sexual relationships [9]. These challenges are closely related to intersex peoples’ medical experiences. Medical intervention on infants’ intersex traits is long-standing. Nowadays, non-medically necessary genital surgery is contested for breaching human rights to bodily integrity and self-determination [10]. However, the practice still happens in the Netherlands [11], whereas in other countries such as Malta, Greece, Spain, Iceland, and Portugal, the practice has been banned. Medical intervention is significant to the experiences of many intersex people, allotting medicine an institutional role in the care and consideration of the intersex community, specifically in the reinforcement of traditional gender roles [12,13]. Furthermore, narratives of intersex people depict how experiences of systematic pathologization of intersex bodies were negatively internalized and affected the sense of self, family function, and social agency [14]. In the meantime, the claimed positive effect of surgery on sexual functioning, psychological consequences, and sexual well-being, when compared with no surgery in people with a DSD condition, is very uncertain [15]. 

Moreover, advised secrecy and stigma impact how intersex people perceive themselves and are perceived by others. Although the stigma, in principle, refers to the intersex or DSD characteristics, it is primarily the undesired effect of ‘being different’ according to others, which harms the self-image and confidence of intersex people [16,17]. As being intersex is often not recognizable from one’s appearance, it requires active disclosure. Even though being able to talk openly about being intersex is beneficial to one’s mental health [18], it does take a lot of courage and constant assessment of whom to trust, as being intersex makes one vulnerable to misunderstanding, stigma, and exclusion [19]. A substantial group of intersex individuals experience psychiatric problems, including anxiety and depression [20,21]. Self-esteem, satisfaction with care, body dissatisfaction, and experiences of shame were associated with psychiatric symptoms. The researchers conclude that developing positive self-esteem and a positive body image is challenging for intersex people, and multidisciplinary care should involve specialized mental health support [20]. 

### 1.1. Sexual and Relational Development

Although sex research regarding intersex individuals tends to focus on sexual (dys)function, a shift is happening toward more positive aspects such as satisfaction and well-being [22,23] and on intersex peoples’ accounts of their sexual experiences [24,25]. A Dutch comparative study explored whether intersex youth differed from non-intersex youth in reaching romantic and sexual milestones, which included ‘falling in love’, ‘kissing with tongue’, ‘dating’, ‘masturbation’, ‘romantic relationship ’, ‘genital caressing’, and ‘oral sex/penetration’ [26]. For each milestone, individuals were asked if they ever experienced it (yes/no) and at what age (in years). Their study showed that although many intersex individuals reached every romantic and sexual milestone, they were significantly older when reaching these milestones. Furthermore, a higher age when reaching several romantic and sexual milestones was correlated with lower self-esteem, lower satisfaction with sexual life, and lower sexual frequency at follow-up. Research dedicated solely to intersex intimate relationships is scarce [27]. Intersex individuals frequently face dating anxieties due to concerns about rejection, personal disclosure, and external appearance. The stigma intersex people face daily, given their genital, gonadal, and sexual forms, heightens the challenges that all individuals face in dating and partnerships [27].

Most of the challenges mentioned above are especially prevalent during adolescence when relational, sexual, and identity development is vital. For example, many young intersex individuals are reluctant to enter into a romantic or sexual relationship because they are uncertain about a new partner’s reaction to being intersex. Reasons for this are that certain forms of sexual behavior are not physically possible, or the social environment problematizes their sexual development [16]. 

Although the above-described studies address some aspects of or explore the sexual development of intersex people, in-depth knowledge about the relational and sexual development of young intersex individuals is still lacking. Specifically, knowledge about factors influencing positive sexual development is absent. More profound knowledge is crucial if we want to support young intersex persons to enjoy sexuality and relationships. For this purpose, we conducted the study presented in this article. 

### 1.2. Current Study

Given the limited pre-existing knowledge on the subject, it was deemed essential to approach the topic more in-depth. Therefore, we chose to answer the research questions with qualitative research, enabling a focus on the experiences of intersex persons. Our goal was to understand the relational and sexual development of young intersex individuals and the factors and actors that influence their relational and sexual development. We formulated the following research questions:

1. How do young intersex persons experience their relational and sexual development?
2. What factors, actors, and life events of young intersex persons contribute to positive relational and sexual development, and what factors are barriers to achieving this?
3. What support needs do young intersex persons have?

The research is approached primarily from the perspective of life course theory [28]. This theory works from the assumption of lifelong development and considers the effect of events earlier in life in shaping current experiences [29]. The biopsychosocial context of development is a vital element in contemporary life course theories [30]. As described above, for young intersex people, their life course contains many specific experiences, such as medical treatments, being diagnosed, and experiences with social stigma. Starting from the life course perspective makes the influence of these experiences visible in the stories young individuals tell. 

Several theories of identity formation emphasize (early) adolescence as an important stage of self-development, albeit in different ways. Examples include the psychoanalytic identity theory (1968) by Erik Erikson [31] and the cognitive psychology approach to the realization of one’s own sex and those of others [32]. We agree with the importance of adolescence as a formative phase and the emphasis placed on interaction in both theories. However, as discussed by Enny Das et al. (2023), in these identity theories, the development of identity is often seen as a linear process with a stable final stage [33] rather than assessing its complex and ongoing nature. Life course theory, then, emphasizes ongoing development throughout the whole life and allows for a more open approach. Moreover, this research does not look at identity formation but at relational and sexual development and the experiences that are formative therein. An informing approach here is the model of sexual agency, in which Cense (2019) describes how young people develop by navigating across different worlds, in social, moral, and narrative connections. Normativity is part of this, but the available narratives and the possible imaginaries play a crucial role as well [34], emphasizing the fundamental relationality with the environment. Of course, identity development occurs in relational and sexual development and in our research. We approach this as an ongoing interactive and performative process [35,36]. 

This leads us to Judith Butler’s theory of heteronormativity (1993) and sexual script theory [37]. Although the primary focus is on the life course perspective, the research is also informed by these theories. Sexual script theory assumes that people form mental representations (scripts) about what sexual behavior—when and with whom—is ‘appropriate’, what effects and reactions to it can be expected, and how one should feel about it [38]. These scripts are based on the cultural scripts available, especially when someone has little experience in a particular area [38]. These cultural scripts are not expected to be appropriate for people whose bodies do not conform to normative definitions of male and female [39]. Butler describes in her extensive discussion of (hetero)normativity how experiences, identities, and events are not only strongly shaped and constructed by heteronormativity but also contribute to it [40]. For example, when applying this to intersex, by keeping what does not fit within the norms invisible, the norm is not challenged but repeated (re-iteration). Due to limited space, we do not zoom in on these approaches in this article. However, it is interesting to note how several participants shared experiences illustrating the reiteration of heteronormative scripts during their treatment trajectories and how these were internalized and thus determined their sexual experiences later. Others reflected on the words and narratives they had to learn to make their stories their own, to reclaim this part of their lives. The sexual script and life course theories both greatly value the actor’s perspective. People partly ‘write’ their scripts by adapting, shaping, and modeling them. In doing so, they partially determine their life course. At the same time, including Judith Butler’s theory of heteronormativity, the difficulty of breaking with internalized scripts is emphasized.

Lastly, the concept of minority stress is important in this research, as it focuses on the experiences of a marginalized group. Minority stress is the stress that intersex people or others experience because of being part of a marginalized group and encountering stigma, discrimination, and prejudice. Part of this stress includes the discrimination, rejection, intimidation, and violence that takes place in the environment on the one hand, and on the other hand, the thoughts, feelings, and behaviors one develops in response to this. These responses are, among other things, based on developed expectations and internalization [41,42]. To thoroughly understand minority stress and its effects, it is essential to consider intersectionality, in which different discrimination grounds and privileges are always seen in the context of each other. In other words, one’s position in society is never determined only by being intersex, for example, but also by skin color, gender identity, expression, abilities, class, etc. [43].

## 2. Methods 

### 2.1. Research Design 

**Figure 1:**
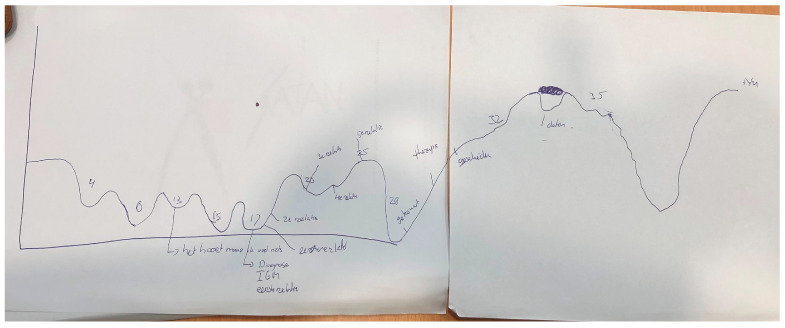
Lifeline of a participant in this study, showing, among others things, the emotional impact of the diagnosis at the age of 17 and the positive effects of being in different relationships between 20 and 25. At the age of 13, the participant wrote, ‘It should be, but I feel nothing’, referring to the idea of having to fall in love with someone but not experiencing these feelings.

In-depth semi-structured interviews were used to invite the participants to share their life stories with us. This approach allowed for flexibility and extended answers where necessary. The interview consisted of two parts. First, participants were asked to draw their lifelines. On the X-axis, we asked them to write their ascending age, starting from birth. On the Y-axis, the level of happiness was the subject. See Figure 1 for a lifeline drawn by a participant in this study. Drawing a lifeline is a narrative technique used as a tool to gain insight into relational and sexual development and other relevant life events [44]. The lifeline helps the interviewee recall past events and allows for self-determination of what is essential in this and what can be shared. In addition, it helps the interviewer organize the information over time and gain insight into the participant’s whole life story. In the lifeline, traumatic events can be revealed, and these can trigger emotions of grief and despair. As we anticipated that telling their story could be stressful for participants, we actively offered all participants to have two conversations with a psychologist/sexologist after the interview. This psychologist was experienced in counseling intersex persons and worked independently from any of the medical institutions where traumatic events could have taken place. Three participants made use of this opportunity. 

The second part consisted of a semi-structured interview using a topic list (see Table 1) based on the research questions and in consultation with the supervisory committee, which included researchers from various fields with experience in intersex research. The Ethics Committee of the Faculty of Social Sciences of Utrecht University (FETC) approved this study prior to commencement. The committee stated that this research project does not fall under the regulation of the Medical Scientific Research Act and, therefore, does not require approval by a Medical Ethics Committee. 

### 2.2. Sample

Participants were recruited through the website of NNID, the Netherlands organization for sex diversity, and their social media channels, via various patient associations, and through personal contact with NNID staff. The patient organization DSD Netherlands, NVACP, Turner Contact, the Dutch Klinefelter Association, and the MRKH Foundation disseminated a call to their members through personal contact, social media channels, and newsletters. Extra efforts were made to recruit male and non-binary intersex people, as these groups are often under-represented in studies concerning intersex people [9].

Ultimately, 18 intersex people between the ages of 18 and 38 participated in this study (see Table 2). The choice for the somewhat older age group—and to collect retrospective data—is threefold. First of all, these young adults are further along in their sexual development and are better able to reflect on it. This older age is especially relevant because of the, on average, later sexual onset of intersex people. Secondly, the choice for the age range 18–38 is essential for the well-being of the participants. Research into the sexual development of intersex persons is a sensitive and complicated subject, which can lead to memories of (sexual) psycho-trauma. Slightly older participants are often further along in coping, reducing the chance of these memories. In the third place, to find enough intersex persons willing to share their experiences on this sensitive topic, we broadened the age range and diversified recruitment methods. 

To protect the anonymity of our participants, we did not include their diagnosis and religious and cultural background in the table below. In the study group, six people were diagnosed with (C)AOS, four people with Klinefelter syndrome, two people with MRKH, two people with 17 beta HSD, and one person with Turner syndrome. In the case of some participants, a specific diagnosis was not possible or not known (*n* = 3). Furthermore, among the participants, nine people were not raised religiously, five had a Christian upbringing but are no longer religious, three were Christian, and one was Muslim. Almost all of the participants are white Dutch. A few participants (*n* = 3) have (an additional) other European backgrounds and some others have, besides a Dutch, also an Asian background (*n* = 2). Many participants did not or did not fully identify as monosexual (*n* = 13), and one person could be described as heterosexual but still preferred not to use this notion. Six persons described their gender identity as (also) non-binary or included intersex in their identity description.

### 2.3. Interviews and Analysis

The interviews took place either online or live at a location of the participant’s choice (at the office of one of the participating organizations—Rutgers and NNID—or at the participants’ home). Three researchers conducted the interviews. As one interviewer is intersex, it was possible to offer participants a choice during the first months of data collection as to whether they wanted to be interviewed by someone who had experiences as an intersex person themselves or instead by an outsider. The insider position has the advantage that the interviewer has a lot of knowledge and a good sense of where sensitive issues lie [45]. It can also be easier for the participant to gain confidence due to shared experiences. On the other hand, the outsider position has the advantage that the interviewer can ask ‘ignorant’ questions and the participant can feel more like an expert on the topic [45]. Due to a change of employers, the ‘insider’ researcher was replaced by a researcher who is not intersex but works within an intersex organization and who had a lot of prior knowledge on the topic. 

The interviews were recorded, transcribed, and anonymized. The transcribed interviews were coded in MaxQDA, starting with deductive thematic analysis following the methodology of Braun and Clarke [46]. The two researchers coded together the first three interviews to create a code tree. This deductive method was followed by a round of inductive coding, in which interview fragments with topics that do not yet fit into the previously established principal codes were taken as the starting point. 

A guidance committee of experts from various organizations critically tested the research approach, providing input throughout the process, including the recruitment strategy, topic list, draft report, and recommendations. Finally, participants received the draft report, enabling them to review the text about their life story and nickname. The report was found to be very relatable. In addition, the preliminary research findings were presented at the annual meeting of DSDnetNL, where medical professionals, behavioral scientists, policymakers, and patient associations were present to verify the results.

## 3. Results

**Figure 2:**
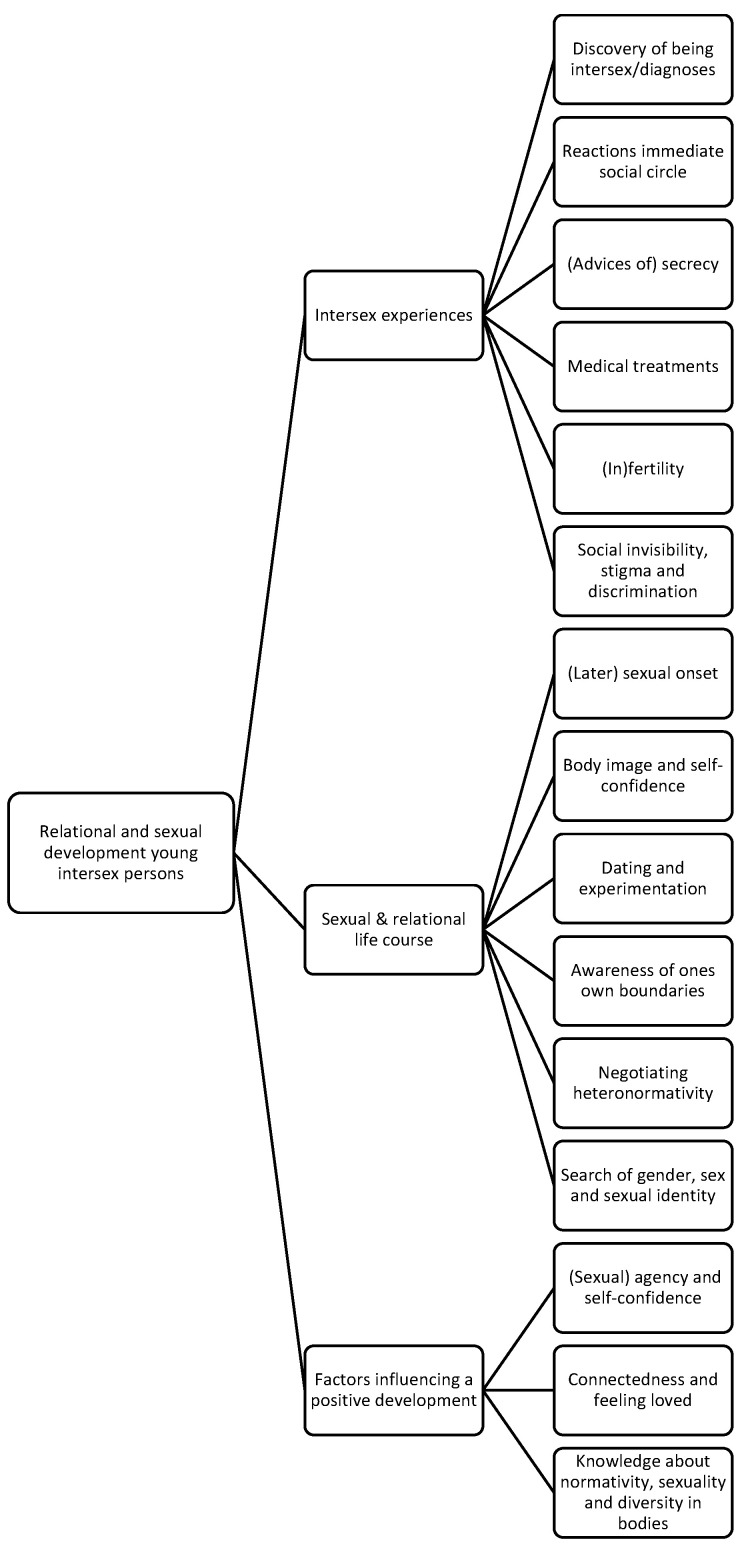
Thematic map of themes.

Based on the research questions, three main themes were identified during the analysis (see Figure 2). These are intersex experiences, the sexual and relational life course, and factors influencing positive development. In the stories participants shared concerning their sexual life course, six recurring tendencies stood out: (1) later sexual onset; (2) negative and/or medicalized body image and low self-confidence; (3) challenges within dating and experimentation, and the importance of intimacy; (4) a compromised ability to be aware of, and the setting of, one’s own boundaries; (5) negotiating norms concerning sex, gender, and sexuality; and (6) strongly related to the latter: the search related to identity in terms of gender, sex, and sexuality. Even though not all participants experienced all six tendencies, their re-occurring presence in the stories of the participants was significant.

It is important to note that both the identified main- and subthemes are inextricably linked; the experiences related to being intersex, often related to medicalization and normativity, have a direct impact on the sexual and relational life course of the participants. The positive factors, then, contain ways of coping with intersex experiences and their impact or are elements the participants identified as missing. 

To understand the participant’s sexual and relational development, it is necessary to first provide an outline of the intersex experiences. These partly overlap with factors discussed in existing literature, as described above, and provide a complete overview of the relevant intersex-related background as described by the participants. After this, we will highlight specific aspects and consequences of these experiences, providing further insight into how they impact sexual and relational development, resulting in the above-mentioned tendencies. Lastly, identified factors influencing positive relational and sexual development will be discussed.

### 3.1. Intersex Experiences

Participants were diagnosed at different times, ranging from the first year of life to high school years. Most participants were told their diagnosis by their parents or doctors during their teenage years. A few participants did not have a medical diagnosis because their parents did not feel it was necessary to have it examined. Apart from receiving a diagnosis, the discovery of one’s own body as “different” also played an important role in the development of body image, in which shame played a central role. Often, this was experienced as a shock. The diagnosis, then, worked as a confirmation of the idea of being “different”, as, for example, Elke (27) describes

*The public health service doctor said when I was 11, ‘Well, you’re small, aren’t you?’ I did not like that very much. Then I had to go to the hospital, and Turner came out. In the period before, I was doing just fine, but I did feel different. But then there was a real dip of OK, I am really different too. You have to start processing that.*

The degree to which parents and other family members reacted with shock to the diagnosis greatly impacted the participants; the more shocked or saddened the family responded, the stronger the feeling grew that something was wrong with them.

*Then my grandfather came in, and I had never seen him cry before, and he broke down completely, panicking and in tears, and then I realized, oh, this is very intense, and something is very wrong with me. I actually thought that.*

(Eva, 24)

Other participants mentioned that their parents handled it well by not putting their own emotions first but instead focusing on their child’s response and being supportive or caring. Some parents took a protective stance regarding unnecessary medicalization, which contributed to the child’s later control over optional treatments and a sense of security.

Some participants faced secrecy as parents or doctors withheld information from them. However, the largest group received confidentiality advice, advising them not to tell their social circle. In this, parents mostly followed advice from the hospital. A minority was advised to be (partly) open. For a few, this turned out wrong (it went around the classroom like a piece of news), but most of the time, openness resulted in social support and ensured the participants’ wellness. Withholding information created a breach of trust with doctors or parents and uncertainty about what happened to one’s own body and who could still be trusted. The advice to keep the diagnosis of being intersex secret had a great impact in terms of social isolation, loneliness, and mental health. This impact only seemed to grow over time.

*I felt a lot of frustration; I felt like I could not move freely in the world. I had to keep a lot to myself, of course. Yes, at one point, I just became a little unkind, a little harsh. That was more the effect of that secrecy.*

(Vera, 37)

Present confidentiality advice also limits the perceived space for learning more about intersex (and associated normativity) outside the medical setting, such as the ability to ask and learn from others about this. This limitation is reinforced by the societal “enforcement” of normativity in the form of discrimination or ignorance. For instance, a biology instructor, when asked by a participant about biological possibilities for intersex, indicated that a person would then be born dead.

The diagnosis was often followed by medical examinations and medical and psychological treatments. The treatments that participants described included vaginal examinations, removal of gonads/testes, hormone treatments (growth hormones or hormones to initiate puberty), psychological counseling, vaginoplasty, and other surgeries such as labia correction, testicular prostheses, and removal of breast formation. For some participants, the treatments assigned a sex that later did not match their gender identity. Some participants expressed satisfaction with their medical journey, but many reflected on a painful period.

*I do have a vagina, but I had to stretch it. […] You literally have to create something in your body that is not there. And that cost me a lot, for my pleasure but also for just being okay with my body. And I do think that in retrospect, with that [treatment], it also became physical. With that, it was no longer just my body, which we’re all going to straighten out a little bit with hormones and surgeries, but with that, it became a physical act that kind of symbolized that I was not allowed to be.*

(Lotte, 29)

In the experience of medical treatments, the participants’ sense of self-determination, the degree of invasiveness of the treatments, and a lack of psychological counseling play an important role. Some treatments were experienced as highly traumatizing and, in some cases, even described as (comparable to) sexual assault or rape.

*Then [after physical examinations], they performed keyhole surgery on me against my will. […] I said in the hospital the day of the surgery that I did not want it, but it was already too late. And I experienced that as very traumatic because I did not know exactly what they...just the thought that they were able to do everything with my body, while I was not awake, that there were all these doctors looking between my legs and also afterward that I had pain between my legs […] Now, after a lot of therapy, I also say that that was actually a sexual assault/rape experience. I am not saying that they raped me because they were doctors, but that is how I experienced it.*

(Azra, 36)

In addition to the invasiveness of the treatments, many participants mentioned they felt unsafe during the treatments due to shortcomings in doctor–patient contact. These aspects are important to mention as these feelings reflect the unequal relationship between the patient and practitioner and the limited possibilities for the patient to break this dynamic. Regarding doctor–patient contact, basic things went wrong, such as forgetting someone’s name, giving wrong information (that someone would have their period after all), and appointments not being kept. The effect was that participants felt vulnerable and suspected that information was being withheld (which was actually the case for some participants). In addition, it was not always explicitly mentioned that participants had the final say in their treatment plan, and age-appropriate information was sometimes lacking, as was the case with Azra (36):

*When I was eleven, I was told to start taking hormones. But without any question as to whether I wanted it or why [it was needed]. At that time, I did not know exactly what was going on, only that I could not have children and that there was a mysterious something not present in my body, of which I had no idea what it meant or what it was.*

Furthermore, (repeated) genital examinations could involve carelessness in asking for permission to have co-assistants join. Finally, the many changes in doctors due to, for example, retirement or participants transitioning from pediatrician to adult care, had an impact on the trust relationship. Here, gender and age differences made building a trusting relationship more difficult.

Another part of the struggle with the diagnosis participants mentioned was to cope with infertility. Although not all intersex persons are infertile, for many participants, this topic has a significant impact on (entering into) relationships. When discussing this topic, it is good to realize that the term infertility does not always cover it very well, as intersex people who are called “infertile” might be able to genetically parent in various ways using techniques such as ICSI, IVF, and surrogacy. In keeping with the heteronormative script dominant in our society, infertility led several participants to question the meaning of their lives and to feelings of not being good enough:

*Then it did feel very heavy because I had lost the meaning of life, and I was also like, if I cannot have children, what then makes me “want” my life?*

(An, 18)

Finally, the silence and stigma in society around intersex impacted the effect of the diagnosis. Many participants experienced discrimination and stigma, ranging from hurtful comments to prejudice and discrimination. In part, this stems from a lack of knowledge about intersex, which leads to embarrassing situations both in the immediate environment and with professionals. These include hurtful questions, wrong assumptions, negative comments, and unabashed curiosity. For example, Lotte (29) experienced the consequences of prejudice and lack of knowledge with her therapist:

*Then I was with [a] therapist, and he said, “Oh, okay, so you had a vagina first, and now that becomes a penis, or how does something like that work"?*

Notable are the different areas and places where the participants experienced discrimination and stigma: at work, at school, while dating and in relationships, in sports, within religion, during further education, online, and at a patient association.

### 3.2. Sexual and Relational Life Course of Young Intersex Persons

The medical journey participants went through forcefully impacted their relationship with their bodies and directly affected their sexual development. The medical treatments and examinations often created a distance between participants and their bodies, where the body was objectified and medicalized, and feelings of alienation or dispassion occurred. An experienced lack of self-determination in treatments contributes to distance from one’s body and contributes to dispossession. In particular, the genital treatments and examinations were often described as intense and painful. Various participants who experienced them also highlighted the lack of choice (do I want this) and guidance, particularly for looking beyond the unquestioned heteronormative premise of having to be able to have penetrative sex. These experiences, the dispossession and distance between the participants and their bodies, had significant implications for sexual experiences, as it resulted in experiencing sex like a medical act and, in some cases, in physical (sometimes traumatic) memories of genital treatments surfacing while having sex.

*“I find that sometimes I do lose touch with my body because you just feel so, yeah, it’s all so objectified, it almost doesn’t even belong to me anymore.”*

(Azra, 36)

One of the described tendencies, the difficulty in experiencing and indicating one’s boundaries, is strongly related to this dispossession. The distance from one’s body resulted in difficulty in experiencing one’s own limits, and because in medical trajectories, control over one’s own body was not always present, for some participants, this also led to difficulty in indicating boundaries in sexual settings. As Robin (37) describes, “That means I couldn’t indicate my boundaries because there was so much waltzing over on all sides that, okay, just do it”. Secrecy counseling reinforces this, as it teaches participants never to talk about their bodies and, thus, their boundaries. Finally, internalized norms form an additional factor: the idea of not wanting to fall behind in terms of first-time kissing or sex recurred for several participants as a reason to ignore their boundaries or needs because “it” has to happen sometimes anyway. These norms include ideas about what sex should be like (penis-in-vagina) and what you are obliged to do as a woman, as Vera (37) describes it:

*“It’s kind of like every time the dick is stiff, something has to be done with it, that obligation I felt very much, and otherwise, you’re not fulfilling as a woman”.*

The gendered normativity on what it means to be female or male is an element several participants encountered on their medical journey, where it was constantly emphasized that they were “otherwise a normal woman or man” and that intersex should not be confused with being transgender. To communicate that someone is not “deviant”, this strategy reinforced binary gender ideas and gender bias. For a limited number of participants, parents and medics chose which gender they were assigned. Both the gendered norms and the gender assignments are practices participants perceived as (very) harmful. Although many participants felt comfortable in the assigned gender, they still experienced an inner struggle around being a “sufficient” or “real” woman or man. For participants who did not feel comfortable in the assigned gender, the battle was obviously even harder. 

Interestingly, many participants dealt with the perceived normativity around sexuality and gender by questioning these norms. Not fitting into societal pigeonholes regarding gender led many participants to question their sexuality and gender, as reflected by the high number of participants with an orientation other than exclusively homosexual or heterosexual (11 out of 18). At the same time, some participants perceived they are being intersex, or their “otherness”, as an inhibiting factor. Feelings about deviating from the norm caused them to want to conform to the norm or not stand out even more. For example, one participant conformed to the heterosexual norm, even though the person was aware that they might not just fall for the opposite sex. Also, concerning gender expression, participants started to dress extra feminine so that people would not think they might be “different”. In addition to the high number of participants (*n* = 13) who did not want to pigeonhole themselves in terms of sexual identity or who identified as pan, bi, or questioning, a relatively large group described themselves as non-binary or questioned the binarity of their sex and gender (*n* = 6). A non-binary identity can help with acceptance of being intersex, and feelings of gender euphoria can be part of this. Sometimes gender and sex are combined in self-identification, such as identities as intersex female or cis intersex non-binary.

In addition to the direct impact that experiences can have on relational and sexual development, they also have a substantial effect on this development through their formative influence on the body and self-image. For example, being diagnosed often led to ideas of being abnormal and to feelings of inferiority, shame, and isolation. Medical treatments—including repeated genital examinations, the fact that they were deemed necessary, and the interest of joining co-assistants—would often lead to the belief of being “some kind of freak” and had a strong impact on self-esteem and feelings of shame. Confidentiality advice had similar effects, and the practice of actively keeping intersex hidden magnified this impact even further. Participants indicated that it was the secrecy that caused shame and feelings of being different. Infertility led to the idea of falling short in a relationship or seeing oneself as unsuitable as a future partner. Experiences of discrimination and stigmatization reinforced these ideas of being different and not fitting in. The various experiences often confirmed and reinforced each other’s messages of being “different” and inadequate, causing deep-seated shame. This resulted in far-reaching consequences in terms of self-confidence and mental health. Furthermore, the developed body and self-image had a strongly inhibiting impact on dating for many participants and caused self-constraint. Intimacy was deemed important in countering the impact of a negative body and self-image but was precisely for the same reasons not always possible.

*“Can I fall in love with that person if they know about me? Yes, it’s always very difficult. It’s also very hard for me to explain. It’s just really the feeling of: I’m not enough. So, I also don’t know if I can really allow infatuation. That I think: yes, but then I have myself with it”.*

(Ellen, 18)

Strongly related to this is the later sexual onset described by some participants, which was caused by shame and feelings of being “different”. At play here and in dating was the idea of physically not being able to meet the expected standard—an idea that is particularly inhibiting in spontaneous sexual contact and early sex in dating. Other factors at play are fear of rejection, previous experiences of stigma and discrimination within dating, and, again, the advised secrecy. The latter includes the issue of when one “should” tell people you are intersex. Whereas some participants had practical arguments for being quick to mention that they are intersex (e.g., because sex is sometimes difficult), others seemed almost to have a “confessional obligation”—feeling morally obligated to tell as soon as possible that they are intersex. Participants felt or felt before that they “had” to tell people about being intersex. Otherwise, they would be selling the other person short. This, then, puts pressure on potential and initial contacts, contributing to the later onset. 

### 3.3. Factors Influencing a Positive Relational and Sexual Development

Firstly, positive sexual development involves daring to express sexual desires and indicate boundaries based on a sense of agency and autonomy. As a part of their intersex experiences, many participants have had damaging experiences precisely in this area of the agency, resulting in an alienation from their bodies. As a result, emotional labor is needed to reclaim their bodies and physically feel their bodies. Furthermore, it takes effort to feel that their desires and boundaries matter, including in sexual relationships. Developing sexual self-confidence seems to go hand in hand with becoming more self-confident in general. As participants felt more self-confident, they talked more easily about being intersex. This openness allowed more intimacy, both with friends and partners. 

One of the steps that improved their self-confidence intersex people took was to claim and tell their own stories. For some, this started early on by doing a paper or a talk about intersex at school. That way, they were able to express themselves and teach those around them how to deal with intersex. Some participants shared their stories through (social) media, art, education, or theatre with a broader audience than their immediate social circle. The motivation behind this was to lift the secrecy and work on social acceptance and change. Imre (31) explained that she created a theatre performance about intersex, which contributed to her empowerment and is meaningful to other intersex persons and the wider society.

*“I also literally took it out of me and made something out of it in the context of processing. [...] So, for my own development, but I also want to be meaningful in another way. And now I get so many messages from other intersex persons who feel supported by the performance or one of the media appearances. That also does me good.”*

(Imre, 31)

Interestingly, the content of what several participants told had changed over time from a medical story to a more personal story about who they were. This shift gives more control over what physical details they do and do not want to share with someone else but also offers more focus on their “self” rather than what diagnosis they were given. They had reframed their own story from a new perspective and a different vocabulary. 

Another way of gaining more self-confidence and discovering who you are and what you want is to allow oneself to experiment with sexuality. It must be noted, however, that it also comes with risks, depending on motivation (a participant told about her experimentation, which was, as she put it, possibly a result of a self-destructive side) and the outcome, as it can also lead to negative experiences. However, when listening to their own boundaries and needs, having positive experiences helped them gain self-confidence. An (18): “So now I’m much more open to dating. I’ve been on three dates and am becoming more confident.” Moreover, participants mentioned how their sexual confidence grew over time because of their mental development. Vera articulated how she has conquered her space and how crucial sexual agency is to her sexual pleasure:

*“I did approach that [sex] differently in my current relationship. I just said, ‘Hey, I don’t really know what I want for a while because I’ve never thought about that very much’, so I state my boundaries rock hard every time, and we’ll see where we end up (laughs). And that’s actually been an excellent strategy because it made me feel like I was in control of my own body and my own form of sex for the first time in my life. And I think that’s what everybody needs to have a nice sex life, sexual agency. And that sexual agency, I just never had it. I think that’s ingredient number one.”*

(Vera, 37)

Secondly, being connected and feeling loved emerged as an important factor contributing to positive sexual and relational development. At first, parents are significant to children’s sense of belonging. As mentioned above, the social support of parents and the feeling of being loved just for who you are are essential buffers against the impact of the diagnosis and medical treatments. Participants reflected on the importance of openness around being intersex at home. The few participants who experienced parents concealing the diagnosis from them later felt very disrespected. In addition, parents must think about the child’s right to self-determination and ensure that no unnecessary examinations, medicalization, or interventions are done to prevent the child from developing the feeling of having no control over their own body (dispossession). Here, the magnitude of the impact of repeated physical (and genital) examinations, the presence of co-assistants, having to undress for examinations, the experience of having your genitals examined, and having no say in what happens to your body cannot be underestimated. Participants felt a need for the non-medical perspective of parents and caregivers, in which the child’s well-being is approached from a broader perspective and can be protected. In terms of sexuality, an open attitude of parents and a non-normative sex education contributed to the self-confidence and freedom of choice that children and adolescents experience. Lars (33): “My father said, ‘I don’t care if you come home with a man or a woman, as long as you’re happy.’ So, it was very nice”. Parents can lay an essential foundation of sex education, where diversity is accepted, as well as diversity in bodies and genitals, gender identity and expression, and sexual orientation. This base supports children and youth in being more flexible with social norms and gives them space to be who they are. 

When getting older, other people are becoming more important when it comes to being connected and feeling loved, like friends and partners. In the participants’ stories, many described their difficulties in trusting a partner, allowing intimacy, and trusting that the other person truly valued them and respected their boundaries. When somebody’s self-esteem is low, it is challenging to allow another person to be close. Participants described how they distanced themselves from a relationship or broke up out of fear of rejection and the conviction that they were ‘not enough’.

*“I can’t give you everything you might want or that you deserve. So then I get in the way of myself and actually kind of sabotage it. At some point, when I think, I feel so nice; I feel so good... I think I am not good enough; you deserve someone who is. So then I think: never mind, it’s not me for you. So then, bye. I have had that very often”.*

(Ellen, 18)

At the same time, participants indicated that a loving partner contributed to more self-confidence and self-acceptance.

*“He just loved my body; he didn’t care at all. He was just really okay with how and who I was. He was also the first one with whom I could talk openly about everything, really literally everything. You know, and he was also really just, um, that I said, “I still have to do those surgeries,” and all he said was, “No, you shouldn’t do that because you’re just fine the way you are and you shouldn’t do that,” and he was always very supportive of me and of my being. So then I think I learned a lot about how I am and who I am and that it’s okay to be that way, too”.*

(Azra, 36)

Connecting with the broader LGBTIQ+ community can also be healing as it allows for breaking with taken-for-granted life courses formed by heteronormativity. The feeling of not being alone plays a significant role. Nina (25) said how being part of this community helped her in her quest to deal with infertility: “The realization indeed that there are others with a broader sexuality, who also cannot get a child together with the one they love. That also gave me a kind of encouragement. I felt that I was not alone in this”.

The third factor visible in the life stories is the importance of gaining knowledge about the existing diversity in bodily characteristics, sexual experience, sexual orientation, and relationship forms. The insecurity many participants experienced in multiple areas due to internalized normativity may diminish through this knowledge.

*“I think that in my youth, the late eighties and early nineties, sex happened in a certain way between a man and a woman, and that’s just the way it should be; that was in the Disney movies, in the commercials, but that was also in the sex education at school and in the education of doctors. They also talked about it that way with me, in terms of how I was going to develop as a girl in puberty and how I had to be prepared for my sex life because otherwise, it couldn’t take place in a normal way. So I very much had this idea of, okay, the only way to have a good life is to have a boyfriend or to be married, and I’m not fit for that right now“.*

(Vera, 37)

Insecurities that intersex individuals can initially feel about their bodies may decrease as they become more knowledgeable about the vast diversity that exists in human bodies, including sex diversity and diversity in sex characteristics. The same is true for fear of failure around sex, which can decrease if people know more about the breadth of sexuality and sexual pleasure.

*“People need to learn to see that sex can be experienced and enjoyed in many ways and that penetration is not the only thing at all. Actually, kind of sex education that all vulvas all look different, that penises all look different, that everyone can have different needs”.*

(Imre, 31)

In response to the above-described challenges, for many participants, meeting other intersex people was crucial to their empowerment and connectedness, which was often facilitated by patient associations and sometimes happened through other avenues, such as social media. Jip (36): “I exchange a lot with people who are intersex on Instagram. That’s where I get my support because they often have the same experiences”. It helps against loneliness to hear how others deal with situations, for recognition and acknowledgment, precisely because in intersex people’s immediate social environment, there are (or seem to be) no other intersex people, or secrecy is upheld. As Vera (37) said,

*“You always fall a little bit outside the norm, and you never have people around you who are the same as you that you can mirror yourself to”*.

## 4. Discussion

This qualitative study explores the factors influencing the sexual and relational development of intersex youth, identifying key experiences, impeding factors, and focusing on factors contributing to positive development. This section discusses how the findings can be interpreted in the context of previous literature, addresses the limitations of the current study, and offers recommendations for future research.

The relational and sexual development of young intersex persons is affected by many different factors. Several factors have to do with experiences people have as a result of being intersex: experiences with diagnostics and medical procedures, (advice of) secrecy, reactions from their social environment, reduced fertility or infertility, stigma, discrimination, and invisibility (including anticipating these occurrences, i.e., minority stress). In these experiences, societal norms of sexuality, gender, and sex play a formative role, for example, when secrecy is advised—in this way, what would challenge the norm is concealed—or when insecurities concerning infertility result from the inability to fulfill the normative picture of becoming a parent in a traditional way. As a result, these experiences, along with the consequential feelings of shame and being perceived as “different” or a “monster”, can lead to a later sexual onset and challenges within dating and experimentation. This confirms and further illustrates previous studies [26,27]. Internalization of discrimination, stigmatization, and prejudices plays an important role, just like the mechanism of anticipating these experiences, strongly pointing toward minority stress. 

This study shows that the medical environment especially poses the risk of creating barriers to positive relational and sexual development, confirming previous findings on the effects of systemic pathologization of intersex bodies [14] and the place of traditional gender roles [12, 13]. Several kinds of experiences in this environment impact receiving a diagnosis (and the related strong response of close relatives to this diagnosis), undergoing treatments and examinations, and being advised of secrecy. These experiences risk implanting and reinforcing a sense of being “different” and deep-rooted shame and can lead to the dispossession of the body. As noted, norms are formative here as well, as is evident, for example, in “normalizing” medical treatments where the body is adapted to fit within binary and oppositional sex norms. When medically unnecessary and executed without the person’s consent, treatments are deemed human rights violations [10] and are banned in several other countries around the world. 

Not only medicalization itself but also interaction between healthcare professionals and intersex people can be an impeding factor, for example, when heteronormativity is present as an unquestioned framework, when information is withheld, self-determination is not discussed, or when age-appropriate information is lacking. These factors can contribute to a sense of feeling “different”, traumatizing events, and dispossession of the body, creating vulnerability to new transgressive experiences. General mistakes, such as forgetting a patient’s name, many changes of healthcare professionals, or asking insensitive or irrelevant questions (in light of the diagnosis), can also create feelings of unsafety, contributing to the experiences mentioned above. 

At the same time, these risks reflect where the medical environment can make a difference in preventing damage and promoting self-confidence and a positive body image by focusing on freedom of choice, refraining from unnecessary medical treatments and physical examinations at an early age, providing respectful treatment, and non-normative counseling that pays attention to social pressures. Moreover, providing sexological and psychosocial counseling can play a vital role, as other scholars found as well. In their study, Berger, Ansara, and Riggs found that for most intersex people, sex education as minors was not relevant to their bodies [24]. Their study also showed that some participants noted that they enjoyed other forms of intimacy, which highlights the importance of focusing on intimacy for intersex people beyond simply ‘penetration’. Given the lack of focus on intersex people in school sex education, it is essential that healthcare professionals can fill this gap when needed. Howe (2021) underlines how healthcare professionals can be supportive when they raise the issues that may trouble intersex people: “sexual intimacy; fertility, including carrying a child; and disclosing to others that they have DSD” [47]. When moving away from preconceived notions of what is ‘normal’ in sex, healthcare providers can encourage intersex persons to explore their existing capacity for sexual relations and enjoyment [48]. 

This study highlights three factors that positively affect the sexual development of intersex people: (1) gaining (sexual) agency and self-confidence, (2) connectedness and feeling loved, and (3) gaining knowledge about normativity, sexuality, and diversity in bodies. Many different actors influence these three enabling factors, including, of course, intersex persons themselves. The life stories of our participants show how intersex persons are active in developing themselves, gaining knowledge, making meaningful connections, and becoming more confident. They achieve this by claiming and telling their own intersex story, being active as an activist or in education, connecting with the (LGBTQ) community, moving away from (hetero)normative codes about gender and sexuality, and expressing themselves through art or work. Their social environment, parents, family, friends, and partners can support intersex youth in this path of self-acceptance, self-confidence, and daring to enter into relationships by appreciating intersex people for who they are, encouraging them to take new steps, being sensitive, respecting boundaries, and thinking non-normatively. 

This study has provided information about a very diverse group. It would be beneficial to conduct follow-up research on the various experiences that are diagnosis- or experience-specific (such as “syndrome” discrimination, physical limitations resulting from specific diagnoses such as Turner and Klinefelter, and the impact of various medical treatments). Furthermore, research on culturally and religiously specific types of support and experienced discrimination would enable providing tools for spiritual and social care workers as well. In addition, our study’s target population was people aged 18 to 38. Because older participants appeared to be well capable of extensive reflections on their earlier relational and sexual development, older participants could be included in a subsequent study. The later sexual onset discussed and the fact that older (outside the scope of this study) intersex persons also addressed this upon reading our call give a good reason for this. Furthermore, this and a larger group of participants would enable analyzing different experiences in different age groups, patterns based on sex/gender assignment, and different experiences depending on the age of disclosure of being intersex. Moreover, some of the experiences described show, to a certain extent, overlap with the experiences of other groups, such as people with disabilities and others encountering “normalizing” treatments and othering in the medical context. Mapping the overlap and differences could provide more insight into the workings and consequences of normative structures.

This research provides insight into factors that play a role in the relational and sexual development of intersex persons. These factors are not static but change over time. For example, medical practices will change due to medical innovation, societal norms, and laws. The social visibility of intersex and, linked to this, the reactions of the social environment are also changing. It is, therefore, important to explore in follow-up research how the bottlenecks currently mentioned by participants evolve in this changing landscape. 

## 5. Conclusions

Our findings show that the current life course of many young intersex individuals is paved with painful obstacles and social challenges that demand great strength to overcome. If we want to achieve healthier and easier relational and sexual development for intersex youth, changes in society, including in the medical sector, are necessary. More space for gender and sex diversity benefits the social acceptance of intersex persons. Parents, healthcare professionals, teachers, and other professionals can all support this tendency by breaking the normative, binary thinking on sex and gender. In the words of one of our participants:

*“Above all, I wish intersex children and young people an easier life, with parents who understand them and not a society that says, “Yes, but there are only boys and girls, and soccer is for boys and ballet is for girls. And I wish that for every child as well.”*

(Robin, 37)

## Figures and Tables

**Table 1:** Overview of topics in the topic list.

Main Topics	Topics
Youth and upbringing	Family climate, values, morality, affection and/or neglect, cultural belonging, religious belonging
Diagnosis	Age, feelings, reactions of their social environment, (medical) consequences, openness/secrecy
Sexuality education	Messages, information, by whom, sufficient? Missing elements.
Puberty and adolescence	How would you describe this period? Did puberty occur spontaneously or through medical involvement? Experience/feelings, the impact of the diagnosis.
Romantic and sexual experiences	Being in love, dating, sexual experiences: feelings, pleasure, experiences of sexual violence. Impact of diagnosis on sexual history.
Sexual and gender identity	How do you identify? Changes over time?
Current sexual and romantic relationships	Description. Satisfied/not satisfied?
Discrimination or exclusion	Experiences, circumstances.
Challenges and resilience	Experiencing problems or mental challenges. Coping strategies. Resilience. Self-acceptance. Social support. Strategies concerning openness/secrecy.
Information and support	Received information and support. Needs. What worked? Or just the opposite? Advice for others, including social environments and medical staff.

**Table 2:** Participants characteristics.

Pseudonym	Age	Gender Identity	Sexual Identity
Ellen	18	Woman	Heterosexual
An	18	Intersex female	Bisexual/questioning
Bas	21	Man	Heterosexual
Eva	24	Woman	Pansexual but hates boxes
Nina	25	Woman	Heterosexual/questioning
Lieke	26	Woman	Heterosexual
Elke	27	Woman	Attracted to men
Jeroen	27	Man	Open minded
Maaike	29	Woman/Non-binary	Bi
Sebastiaan	29	Non-binary	Pansexual
Lotte	29	Cis woman	No label (attracted to people)
Alexander	30	Cis man	Heterosexual
Imre	31	Woman	Bisexual
Lars	33	Non-binary	Pansexual
Azra	36	Woman	Attracted to people
Jip	36	Cis-intersex non-binary	Pansexual
Vera	37	Woman	Heterosexual/questioning
Robin	37	Non-binary	Pansexual

## Data Availability

The data presented in this study is only available on request due to privacy restrictions.
